# Comparative Transcriptomic Analysis Revealed the Suppression and Alternative Splicing of Kiwifruit (*Actinidia latifolia*) *NAP1* Gene Mediating Trichome Development

**DOI:** 10.3390/ijms24054481

**Published:** 2023-02-24

**Authors:** Tonghao Miao, Huaxu Bao, Hui Ling, Pengwei Li, Yiling Zhang, Yan He, Xufan Hu, Chengcheng Ling, Yunyan Liu, Wei Tang, Yajing Liu, Songhu Wang

**Affiliations:** School of Horticulture, Anhui Agricultural University, Hefei 230036, China

**Keywords:** kiwifruit, trichome development, transcriptomic analysis, *NAP1*

## Abstract

Kiwifruit (*Actinidia chinensis*) is commonly covered by fruit hairs (trichomes) that affect kiwifruit popularity in the commercial market. However, it remains largely unknown which gene mediates trichome development in kiwifruit. In this study, we analyzed two kiwifruit species, *A. eriantha* (Ae) with long, straight, and bushy trichomes and *A. latifolia* (Al) with short, distorted, and spare trichomes, by second- and third-generation RNA sequencing. Transcriptomic analysis indicated that the expression of the *NAP1* gene, a positive regulator of trichome development, was suppressed in Al compared with that in Ae. Additionally, the alternative splicing of *AlNAP1* produced two short transcripts (*AlNAP1-AS1* and *AlNAP1-AS2*) lacking multiple exons, in addition to a full-length transcript of *AlNAP1-FL*. The defects of trichome development (short and distorted trichome) in *Arabidopsis nap1* mutant were rescued by *AlNAP1-FL* but not by *AlNAP1-AS1*. *AlNAP1-FL* gene does not affect trichome density in *nap1* mutant. The qRT−PCR analysis indicated that the alternative splicing further reduces the level of functional transcripts. These results indicated that the short and distorted trichomes in Al might be caused by the suppression and alternative splicing of *AlNAP1*. Together, we revealed that *AlNAP1* mediates trichome development and is a good candidate target for genetic modification of trichome length in kiwifruit.

## 1. Introduction

Kiwifruit (*Actinidia chinensis*) is a popular and health-beneficial fruit because of its high vitamin C content and balanced nutrients, including dietary fiber, various minerals, and other metabolites [1,2]. Kiwifruit consumption improves immune, digestive, and metabolic health and even provides anticancer effects [3,4].

Fruit hair (trichome) is an important appearance quality that affects kiwifruit popularity in the market [5]. Some cultivars, such as *A. deliciosa* ‘Hayward’, have long coarse trichomes, which are generally considered a commercial disadvantage [5]. The surface trichomes of ‘Hayward’ could be removed by brushing for customer favor, but brushing accelerates kiwifruit softening and reduces the shelf life [6]. *A. deliciosa* was crossed with *A. arguta*, which has hairless and edible fruit skins, in order to breed a new cultivar with hairless skins [7]. Although micromorphological characters of trichomes have been used to analyze phylogenetic relationships within the genus *Actinidia* [8], little is known about kiwifruit’s genes mediating trichome development.

Trichomes are epidermal protrusions that developed from the surfaces of leaves, stems, flowers, seed coats, and fruits. Trichomes protect plants from insect predation, UV radiation, and excess transpiration [9]. In *Arabidopsis*, the genes mediating trichome development have been extensively identified [10,11,12]. The initiation and development of trichome are majorly regulated by the transcriptional complex involving three types of transcription factors (TFs): the R2R3 MYB, basic helix-loop-helix (bHLH), and WD40 repeat (WDR) protein. In the R2R3 MYB family, GLABROUS 1 (GL1) [13], MYB23 [14], and MYB82 [15] are involved in trichome development and differentiation. The bHLH TFs affecting trichome development include GLABROUS 3 (GL3) [16], ENHANCER OF GL3 (EGL3) [17], TRANSPARENT TESTA 8 (TT8) [18], and MYC1 [19]. TTG1, a WDR gene, mediates trichome differentiation [20]. Some R3 MYBs, which include TRIPTYCHON (TRY) [21], CAPRICE (CPC) [22], ENHANCER OF TRY AND CPC1 (ETC1, ETC2, and ETC3) [23,24], and TRICHOMELESS 1 (TCL1 and TCL2) [25,26], are negative regulators of trichome development. Additionally, phytohormones, miRNA, and ubiquitin/26S proteasome can affect trichome development by directly or indirectly targeting the TFs mediating trichome development [10,12,27,28,29,30]. For instance, the ubiquitin protein ligase 3 (UPL3) is involved in trichome development by mediating proteasomal degradation of GL3 and EGL3 [27].

The ARP2/3-mediated nucleation of actin filaments is involved in trichome development [31,32]. The SCAR/WAVE complex is the major activator of ARP2/3-mediated F-actin nucleating and branching [33]. A defect in Nck-associated protein 1 (NAP1), a subunit of the SCAR/WAVE complex, causes short and distorted trichomes in *Arabidopsis* [34,35]. In soybean, two mutants with shorter and distorted trichome were revealed by map-based cloning to be caused by loss-of-function mutations in *Glycine max NAP1* (*GmNAP1*) [36,37]. These results indicated that NAP1 plays an important role in trichome development.

Transcriptomic analysis has been widely used to discover the genes mediating trichome development in different species, such as tomato [38], cucumber [39], Lilium pumilum [40], and medicinal cannabis [41]. In this study, we analyzed two kiwifruit species (*A. eriantha* [42] with long bushy trichomes and *A. latifolia* [43] with short, sparse trichomes) by second- and third-generation transcriptome analysis. Our results indicated that the expression level of the *NAP1* gene was much lower in *A. latifolia* than that in *A. eriantha*. Meanwhile, we discovered that there is alternative splicing of *NAP1* mRNA in *A. latifolia*, which might be responsible for the shorter trichomes in *A. latifolia*.

## 2. Results

### 2.1. Morphologic Observation of Trichomes in Two Kiwifruit Species

**Figure 1:**
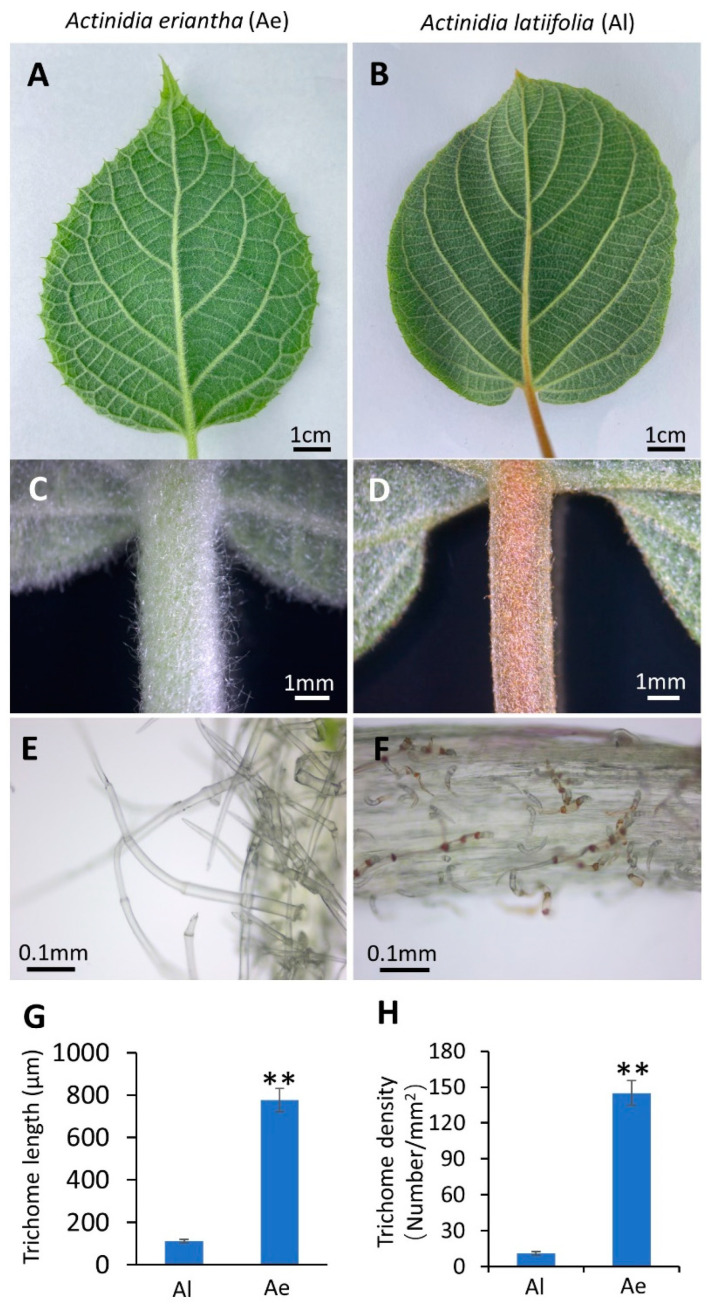
Morphologic observation of trichomes in two kiwifruit species. The mature leaf of *A. eriantha*, Ae for short (**A**), and *A. latifolia*, Al for short (**B**). The petioles of mature leaves from Ae (**C**) and Al (**D**). Trichome of petioles of Ae (**E**) and Al (**F**) was observed in microscope. Trichome length (**G**) and density (**H**) are shown in the mean values ± SD from three replicates. Significant differences were determined by Student’s *t*-test (** indicates *p* < 0.001).

The fruits of *A. latifolia* (Al) are sparsely covered by short trichomes (Figure S1A), while *A. eriantha* (Ae) fruits are densely covered by long intertwining trichomes (Figure S1A), which is consistent with the previous observation [44]. The contrasting distribution patterns between Al and Ae trichomes were also observed in the petiole of mature leaves (Figure 1A–D). The epidermis of Al petiole is attached with short trichomes (Figure 1D,F), and the average length of trichomes is 100 μm (Figure 1G). The trichomes of Ae petioles are much longer than that in Al (Figure 1C,E,G). Additionally, the trichome density in Ae petioles is much higher than that in Al (Figure 1H). It is worth mentioning that Al trichomes are mildly distorted and brown (Figure 1F), while Ae trichomes are straight and colorless (Figure 1E). These observations indicated that the morphologic characterizations of Al and Ae trichomes are quite different.

### 2.2. The Third- and Second-Generation Transcriptome Analysis

Since the time of flowering and fruit setting is quite different between these two species, the climatic conditions will dramatically affect the gene expression if we harvest the fruits at different times to perform the transcriptome analysis, which will obstruct our digging for the differentially expressed genes mediating trichome development. Considering that trichome distribution patterns on the fruits are similar to that on the petiole of mature leaves in two species (Figure 1), we used the petioles of mature leaves from the plants of two species grown in the same field for the RNA sequencing. Because the genome sequence of Al is lacking, the third-generation (full-length) RNA sequencing of mature leaves, including the petioles from Al and Ae, was performed to facilitate gene annotations.

**Figure 2:**
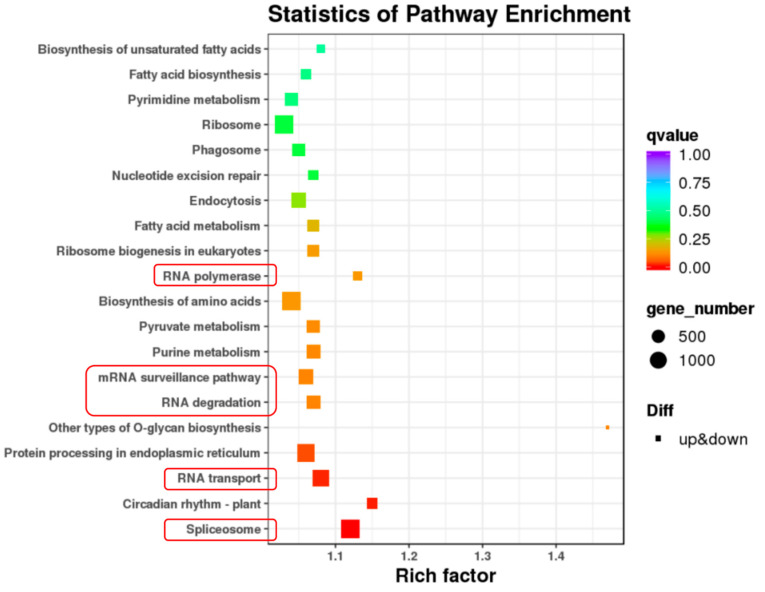
KEGG enrichment analysis of differentially expressed genes. KEGG enrichment of DEGs between *A. latifolia* and *A. eriantha*. The pathways of RNA spliceosome, transport, degradation, surveillance pathway, and polymerase were highlighted by red frames.

As shown in Table 1, we obtained 135,286 and 156,752 high-quality isoforms from third-generation RNA sequencing of Al and Ae, respectively. Among them, there are 126,102 and 93,226 non-redundant transcripts for Al and Ae, respectively (Table 1). These transcript sequences will contribute to gene annotations and further functional characterization. Meanwhile, three biological replicates of the epidermis of petioles samples from Al (Al-q1, -q2, and -q3) and Ae (Ae-q1, -q2, and -q3) were harvested for the second-generation RNA sequencing. The PCA analysis indicated that there is a high correlation among the three samples of each cultivar, suggesting a high repeatability of RNA sequencing results (Figure S2). Next, we discovered 88,270 differentially expressed transcripts (DETs) (listed in Table S1), including 39,879 up-regulated and 48,391 down-regulated transcripts between Al and Ae samples (Table 2). These DETs were also mapped to the genome of ‘White’ [42], which is provided by the kiwifruit genome database (http://kiwifruitgenome.org/, accessed on 3 October 2021) [45], and 12,950 differentially expressed genes (DEGs) were identified (Table 2). KEGG analysis of DEGs indicated that the pathways of RNA metabolisms, including RNA processing (spliceosome), transport, degradation, surveillance, and polymerase (synthesis), were enriched (Figure 2). No pathway of trichome development has been identified in KEGG enrichment.

**Figure 3:**
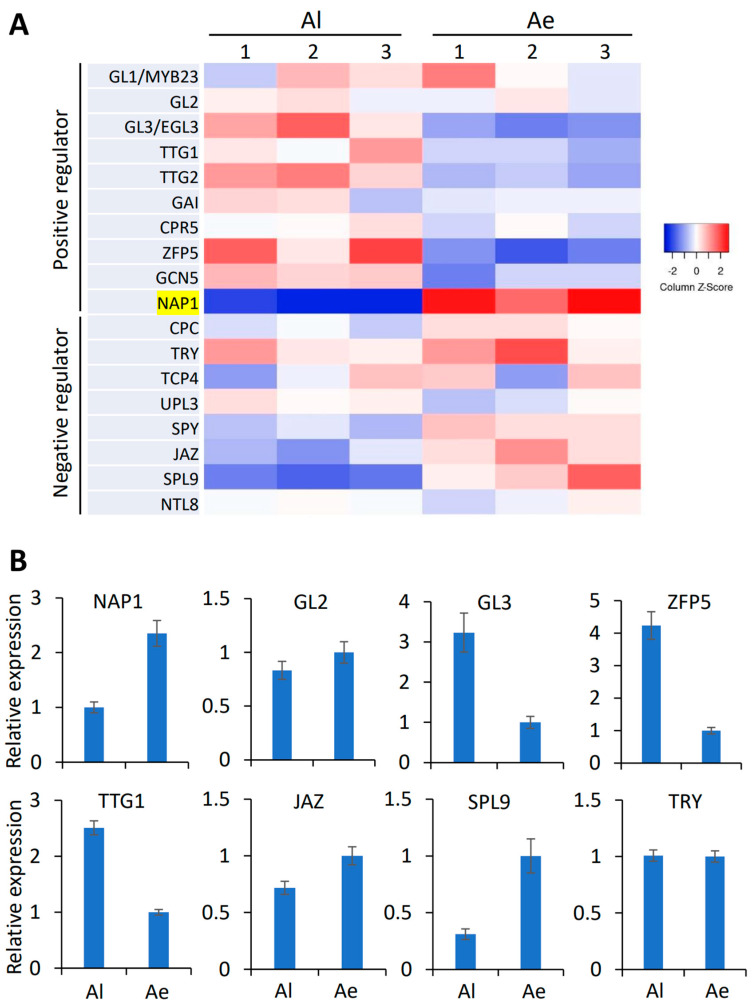
The expression levels and qRT−PCR verification of trichome-regulating genes in Al and Ae. (**A**) The expression levels of positive and negative regulators of trichome development in three replicates of RNA sequencing. (**B**) qRT−PCR analysis of these genes in Al and Ae. The relative expression levels are shown in the mean values ± SD from three biological replicates.

Next, we screened out the homologous genes of trichome development-regulating genes in *Arabidopsis* from all the DEGs and compared their expressions between Al and Ae (Figure 3A). Unexpectedly, the expression levels of most genes encoding a positive regulator of trichome development in Al, such as GL3, TTG1, TTG2, Constitutive Expressor of Pathogenesis-Related Genes5 (CPR5) [46], Zinc Finger Protein5 (ZFP5) [47], and GENERAL CONTROL NON-REPRESSED PROTEIN5 (GCN5) [48], are higher than that in Ae (Figure 3A) while most of the genes encoding negative regulators, such as CPC, SPINDLY (SPY) [28], Jasmonate ZIM (JAZ) [29], and SQUAMOSA PROMOTER BINDING PROTEIN-LIKE 9 (SPL9) [30], are down-regulated in Al but up-regulated in Ae (Figure 3A). These results were opposite to our expectation since Al with fewer trichomes are supposed to down-regulate the positive regulators or up-regulate the negative regulators. The expression level of UPL3, a negative regulator, appeared to be lower in Ae than in Al (Figure 3A), which requires further verification. Only one positive regulator, NAP1, as highlighted in yellow in Figure 3A, showed low expression in Al but a high expression in Ae, which is consistent with the phenotypic observation. Our qRT-PCR analysis confirmed that the expression patterns of *NAP1* and other regulators of trichome development are consistent with the results of RNA seq (Figure 3B). These results indicated that down-regulated *NAP1* in Al might contribute to the phenotype that Al has short, sparse trichomes.

### 2.3. Alternative Splicing of AlNAP1 mRNA

**Figure 4:**
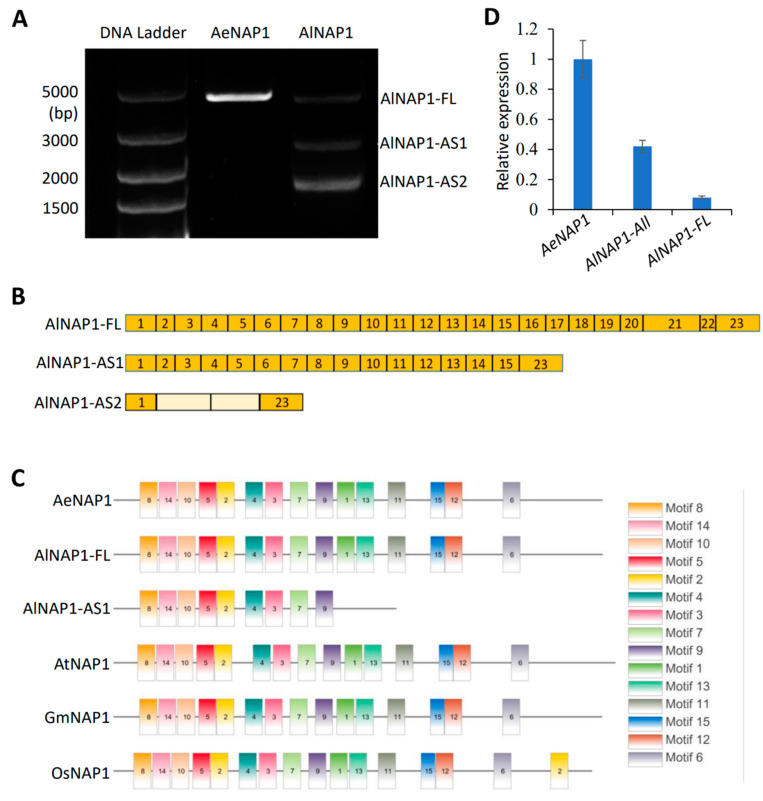
The alternative splicing of *AlNAP1* mRNA. (**A**) The agarose gel analysis of RT-PCR products of AeNAP1 and AlNAP1, including full-length AlNAP1 (AlNAP1-FL) and two spliced transcripts (AlNAP1-AS1 and AlNAP1-AS2). DTZ79_14g00610 is the accession number of *AeNAP1* in the genome sequence. (**B**) The exon structures of AlNAP1-FL, AlNAP1-AS1, and AlNAP1-AS2. The exon is shown in orange, and the intron is shown in pale yellow. (**C**) Motif analysis of AeNAP1, AlNAP1-FL, AlNAP1-AS1, AtNAP1 (Arabidopsis), GmNAP1 (soybean), and OsNAP1 (rice) by MEME Suite website. (**D**) qRT-PCR analysis of AeNAP1, AlNAP-FL, and AlNAP1-All (including AlNAP1-FL, -AS1, and -AS2). The relative expression levels are shown in the mean values ± SD from three biological replicates.

To clone the *NAP1* gene in kiwifruit, we used the samples for RNA-seq as a template and the same pair of primers targeting the 5′ and 3′ UTRs of both *AeNAP1* (DTZ79_14g00610, accession number of Ae genome) and *AlNAP1* to perform RT-PCR. The results showed that Ae has a clear band with the expected size (Figure 4A), while Al has two alternatively spliced bands, named *AlNAP1-AS1* and *AlNAP1-AS2*, in addition to the full-length band *AlNAP1-FL* (Figure 4A). These bands were cloned and analyzed by Sanger sequencing. The results indicated that the encoding sequences of *AeNAP1* and *AlNAP1-FL* are almost the same except for two SNPs causing the substitution of two amino acids (Figure S3).

We identified the 23 exons of *AlNAP1* by comparing its cDNA sequence with the genome sequence of AeNAP1 Locus (Figure 4B). The sequencing results of *AlNAP1-AS1* and *AlNAP1-AS2* indicated that *AlNAP1-AS1* is short of the exons from 16th to 22nd, while *AlNAP1-AS2* lacks most exons but remains the 1st and 23rd exons linked with two introns (Figure 4B). The motif-based sequence analysis [49] indicated that AeNAP1 and AlNAP1-FL contain 15 conserved motifs that are present in all NAP1 proteins from Arabidopsis, rice, and soybean (Figure 4C). *AlNAP1-AS1* encodes a short protein lacking the conserved motif 1, 6, 11, 12, and 15 (Figure 4C), while *AlNAP1-AS2* encodes a very short peptide having no similarity with NAP1, suggesting that AlNAP1-FL might have the conserved function in trichome development while the AlNAP1-AS1 and -AS2 should lose the function. The qRT−PCR indicated that the expression level of all transcripts of *AlNAP1,* including *AlNAP1-FL*, *-AS1*, and *-AS2* is about 40% of that of *AeNAP1* (Figure 4D). The expression of AlNAP1-FL is only 8% of that of *AeNAP1* (Figure 4D). These results indicated that the alternative splice of *AlNAP1* further decreases the level of functional proteins. Moreover, the alternatively spliced bands of *NAP1* were also amplified by RT−PCR from the epidermal tissues of Al young fruits but not from that of Ae fruits (Figure S1B), suggesting that the alternative splice of *AlNAP1* occurs in both leaves and fruits.

### 2.4. Complementation of Arabidopsis nap1 Mutant Was Achieved by AlNAP1-FL but Not by AlNAP1-AS1

**Figure 5:**
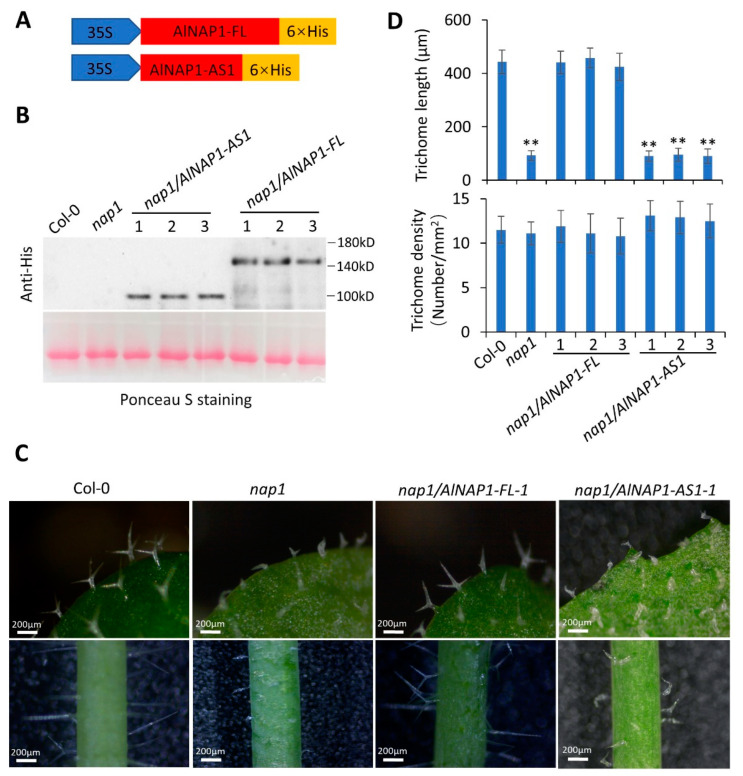
The complementation of *nap1* mutant was achieved by *AlNAP1-FL* but not *AlNAP1-AS1*. (**A**) The constructs of vector overexpressing AlNAP1-FL and AlNAP1-AS1. (**B**) Western-blot analysis of Col-0, *nap1* mutant, three independent transgenic lines of *nap1/AlNAP1-AS1* and *nap1/AlNAP1-FL* by using anti-His antibodies. (**C**) The observation of leaf and stem trichomes of Col-0, *nap1* mutant, *nap1/AlNAP1-AS1-1*, and *nap1/AlNAP1-FL-1*. (**D**) Trichome length and density of leaves of Col-0, *nap1* mutant, *nap1/AlNAP1-AS1*, and *nap1/AlNAP1-FL*. More than 20 trichomes for each line were measured and showed in the mean values ± SD. Significant differences were determined by Student’s *t*-test (** indicates *p* < 0.001).

To verify the function of *AlNAP1* in trichome development, we tried to express *AlNAP1-FL* and *AlNAP1-AS1* in the mutant *nap1-2* (SALK_014298, *nap1* for short) of *Arabidopsis* [34]. A his-tag was fused to the C terminus of AlNAP1-FL and AlNAP-AS1, respectively, and the fused genes were driven by the CaMV35S promoter (Figure 5A). The *nap1* mutant was transformed by the agrobacteria containing the construct of 35S:: AlNAP1-FL-His and 35S::AlNAP1-AS1-His, respectively. Three independent transgenic lines for each construct were identified by Western blot analysis using anti-His antibodies (Figure 5B). The *nap1* mutant showed shorter and distorted trichomes [34] compared with the Col-0 (Figure 5C,D). Three independent transgenic lines of *nap1/AlNAP1-FL* (−1, −2, and −3) showed similar phenotypes in that their trichomes are long and straight (Figure 5C just showed the trichomes of *nap1/AlNAP1-FL-1*). The trichomes of *nap1/AlNAP1-FL*-1 are much longer than that of *nap1* and indistinguishable from that of Col-0 plants (Figure 5D). However, three transgenic lines of *nap1/AlNAP1-AS1* (−1, −2, and −3) showed short and distorted trichomes (Figure 5C just showed the trichomes of *nap1/AlNAP1-AS1-1*), which are as short as that of *nap1* mutant (Figure 5D). The trichome density of the *nap1* mutant is not altered by *AlNAP1-FL* or *AlNAP1-AS1* overexpression (Figure 5D). These results indicated that *AlNAP1-FL*, but not *AlNAP1-AS1*, rescued the defects of trichome development in the *nap1* mutant.

## 3. Discussion

The trichomes protect plants from mechanical damage, insect predation, UV radiation, and excess transpiration [9]. In kiwifruit, trichomes might play a role in the resistance to bacterial canker caused by *Pseudomonas syringae* pv. *Actinidiae* [50]. Commercially, trichomes affect the kiwifruit popularity in the market [5]. Although brushing with commercial brushes has been practically used to remove the surface trichomes of kiwifruit, brushing promotes kiwifruit softening and reduces the shelf life [6]. Therefore, it is better to breed new kiwifruit cultivars with shorter and fewer trichomes or genetically modify the popular cultivar by targeting the key genes mediating trichome development. The genes regulating trichome development have been extensively identified in *Arabidopsis* [10,11,12]. To the best of our knowledge, no such gene has been characterized in kiwifruit.

In this study, we observed that Al has short, mildly distorted, and brown trichomes (Figure 1F), while Ae has long, straight, and colorless ones (Figure 1E). The trichome density in Al petioles is much lower than that in Ae (Figure 1H). By the second- and third-generation transcriptome analysis, we revealed that the mRNA levels of most DEGs mediating trichome development are contrary to the observed phenotypes of trichome (Figure 3). Only *NAP1* expression, which is lower in Al than in Ae, is consistent with the trichome phenotypes (Figure 3). NAP1, a subunit of the SCAR/WAVE complex, is required for the activation of the Arp2/3 complex, which is a crucial regulator of actin nucleation and branching. A defect on *NAP1* gene causes short and distorted trichomes in both Arabidopsis [34,35] and soybean [36,37], although the underlying mechanisms of regulating trichome development remains largely unknown. In our study, the alternative splicing of *AlNAP1* mRNA occurs in both leaves and fruits of Al but not in that of Ae (Figure 4 and Figure S1B). Sanger sequencing indicated that two spliced mRNA, *AlNAP1-AS1* and *AlNAP1-AS2*, lost 7 exons and 21 exons, respectively (Figure 4B). *AlNAP1-AS2* encodes a totally different protein that has no similarity with NAP1. *AlNAP1-AS1* encodes a short protein lacking the conserved motif 1, 6, 11, 12, and 15 (Figure 4C) and cannot rescue the defects of trichome development in mutant *nap1* of *Arabidopsis* (Figure 5), suggesting that AlNAP1-AS1 is not a functional protein. The alternative splicing further down-regulated the expression level of *AlNAP1-FL* (Figure 4D), which has been verified to have functions in regulating trichome development (Figure 5). Defects on *NAP1* genes cause shorter and distorted trichome in *Arabidopsis* [34] and soybean [36,37]. Therefore, we speculate that the suppression and alternative splicing of *AlNAP1* is responsible for the short, mildly distorted trichome of Al. Considering that the trichome densities in *nap1* mutant and *nap1/AlNAP1-FL* plants are not altered compared to that of Col-0 (Figure 5), we believe that low trichome density in Al has nothing to do with the *AlNAP1* gene. Of course, verification of our speculation requires further investigations, such as genetic modification of the *NAP1* gene in Al, which is currently not practicable because the method of Al transformation is not established yet.

As for the reason why alternative splicing of *NAP1* exists in Al but not in Ae, we revealed that the genes belonging to spliceosome were most significantly enriched in our transcriptomic analysis (Figure 2). Differentially expressed spliceosome genes in Al might contribute to the alternative splicing of *AlNAP1*. To date, no spliceosome gene has been demonstrated to participate in trichome development. It will be interesting to figure out which spliceosome gene plays a key role in the processing of *AlNAP1* in the future.

Among the negative regulators identified by our transcriptome, *UPL3* appeared to have lower levels in Ae than in Al (Figure 3A). UPL3 is an E3 ligase mediating ubiquitin/26S proteasome-dependent degradation of GL3 and EGL3 [27]. Overexpression of *GL3* significantly increased the trichome number in *Arabidopsis* [16] and even had a stronger effect on trichome density in the *upl3* mutant [27]. Therefore, we speculate that the lower expression of *UPL3* in Ae might cause the increased protein abundance of GL3, which might be responsible for the high trichome density in Ae.

Together, our results indicate that the shorter and distorted trichomes in Al might be caused by the suppression and alternative splicing of *AlNAP1*, which can be a potential target for genetic modification of trichome length in kiwifruit.

## 4. Materials and Methods

### 4.1. Plant Material and Growth Conditions

The kiwi species “*Actinidia eriantha*” and “*Actinidia latifolia*” were grown on a kiwi plantation at Anhui Agricultural University. Hefei, China. The leaves and petioles of “*Actinidia eriantha*” and “*Actinidia latifolia*” with similar development periods and states were cut down and immediately frozen in liquid nitrogen and stored at −80 °C.

The *Arabidopsis nap1* mutant strain SALK_014298 was ordered from the AraShare Science website (www.arashare.cn, accessed on 10 November 2021). Homozygous plants were identified by the triple primer method, as described previously. *Arabidopsis* Columbia ecotype (Col-0) and *nap1* mutant were sterilized and vernalized for 3 d at 4 °C and then were sown on MS medium for 10 d under 14 h light/10 h dark cycle at 22 °C. Then, *Arabidopsis* seedlings were transferred to a soil mixture of vegetative soil and vermiculite (3:1, *v*/*v*) and grown at a long-day condition (22 °C, 14/10-h light/dark).

### 4.2. RNA Extraction and qRT−PCR Assays

RNA was extracted from the leaves of “*Actinidia eriantha*” and “*Actinidia latifolia*” using an RNA plant plus Reagent kit (TianGen, Beijing, China) and transcribed into cDNA using the PrimeScript^®^ RT reagent kit (Perfect Real Time, TaKaRa). Fluorescent quantitative primers were designed by Primer-BLAST in NCBI (www.ncbi.nlm.nih.gov/tools/primer-blast/, accessed on 25 November 2021). The qRT–PCR was carried out using the CFX connectTM Real-Time System (BIORAD, US), qRT–PCR reactions were as follows: pre-denaturation at 95 °C for 10 min, denaturation at 95 °C for 15 s, annealing at 56 °C for 15 s, extension at 65 °C for 10 s, 40 cycles, relative expression values were analyzed via the cycle threshold (Ct) 2^−ΔΔCT^ method [51]. Three biological replicates for each sample and three technical replicates for each biological replicate. The qRT−PCR primers are listed in Supplemental Table S2.

### 4.3. Cloning of AlNAP1 and AeNAP1

The full-length cDNA of *AlNAP1* and *AeNAP1* were amplified with the first strand cDNA using the forward primer 5′-TCCAACAATCGGCCTTCCCTAC-3′ and the reverse primer 5′-AGGACCAGACCTTGACACAGC-3′, respectively. The genes were amplified by PrimeSTAR^®^ Max DNA Polymerase (Takara, Dalian, CN), and PCR reaction conditions were pre-incubation at 98 °C for 3 min, followed by 35 cycles of 98 °C (30 s), 55 °C (30 s), and 72 °C (5 min), with a final extension at 72 °C for 5 min. The PCR product was recovered, ligated to the pESI-Blunt simple vector, and then transferred into *E. coli*. Recombinant clones were identified by PCR and sequencing.

### 4.4. Vector Construction and Plant Transformation

A His-tag (6×His) was fused to the coding sequence of *AlNAP1-FL* and *AlNAP1-AS1*, respectively, by PCR. The fused genes *AlNAP1-FL-His* and *AlNAP1-AS1-His* were transformed into the expression vector pCAMBIA1302. The constructed vectors *35S::AlNAP1-FL-His* and *35S::AlNAP1-AS1-His* were transformed into *Arabidopsis nap1* mutant strain using the floral dip transformation method as described previously [52]. Transgenic lines were then screened with 60 mg/L hygromycin. Two independent transgenic plants for each vector were identified and used for further experiments.

### 4.5. Sequence Alignment and Protein Domain Analysis

Sequence alignments were performed using ApE software, a phylogenetic tree was performed using the neighbor-joining method by the MEGA (ver. 7.1), and protein domains were analyzed using the NCBI Conserved Domain Search website (www.ncbi.nlm.nih.gov/Structure/cdd/wrpsb.cgi, accessed on 8 January 2022). The motifs of the *NAP1* gene were further analyzed using the MEME—MEME Suite website (meme-suite.org, accessed on 10 January 2022) [49].

### 4.6. Third- and Second-Generation Transcriptome Sequencing

Since the time of flowering and fruit setting is quite different between these two species, the climatic conditions will dramatically affect the gene expression if we harvest the fruits at different times to perform the transcriptome analysis, which will obstruct our digging for the differentially expressed genes mediating trichome development. Considering that trichome distribution patterns on the fruits were similar to that on the petiole of mature leaves in two species, we used the mature leaves and the epidermal tissues of the petiole for third- and second-generation transcriptome analysis, respectively. For the third-generation transcriptome, we just had one sample, which is a mixture of three leaves from different trees. Total RNA was extracted from the mature leaves (including petioles) of “*Actinidia eriantha*” and “*Actinidia latifolia*” and used for third-generation transcriptome analysis. For the second-generation transcriptome, we have three samples (biological replicates) for each specie, which means that three petioles were detached from three different trees. Total RNA was also extracted from epidermal tissues of petioles and used for second-generation transcriptome analysis. The RNA sequencing and analysis were performed by Biomarker biotech Co., Ltd. (Qingdao, China). Briefly, after total RNA was extracted, eukaryotic mRNA was enriched by magnetic beads with Oligo (dT) and then was randomly interrupted by fragmentation buffer. One-stranded cDNA was synthesized from the interrupted mRNA template using six-base random primers. Then, the second cDNA strand was synthesized by adding buffer, dNTPs, RNase H, and DNA polymerase I. The resulting double-stranded cDNA was purified using AMPure XP beads, and the terminal was repaired, added with A tail, and connected with the sequencing connector, and then AMPure XP beads were used to select the fragment size, and the cDNA library was obtained by PCR enrichment. The constructed library was sequenced using the Illumina platform to generate 150 bp double-ended reads; the total read is above 6G bp. The RNA sequencing data were obtained from three biological replicates, and PCA analysis was used as an assessment indicator of biological repeat relevance. PCA analysis was performed using the base package stats in R (v3.1.1), and visualization was performed using the ggplot2 package (v1.0.1). The data were obtained from three biological replicates, use of Spearman’s correlation coefficient R as an assessment indicator of biological repeat relevance. The expressed genes were analyzed; log2 fold change values > 1 and log_2_ fold change values < −1 were considered significant. The significantly induced (>2-fold) and repressed (<0.5-fold) genes are listed in Supplemental Table S1.

### 4.7. Protein Extraction and Western Blotting

Approximately 500 mg of *Arabidopsis* seedlings were ground in a 2× Laemmli sample buffer containing 100 mM Tris-HCl (pH6.8), 4% SDS, 0.2% BPB (bromophenol blue), 2% beta-ME, and 20% glycerol. The samples were boiled at 100 °C for 15 min and then centrifuged for 10 min at 12,000 rpm. Protein extracts were separated on a 10% SDS-PAGE and transferred to PVDF blotting membranes as described previously [53]. The *35S::AlNAP1-FL-His* and *35S::AlNAP1-AS1-His* protein amounts were determined by protein gel blotting using anti-His antibodies.

### 4.8. Statistical Analysis

Epidermal samples were photographed under a microscope (E200MV; Nikon, Nanjing, China), and the length and density of epidermal fur were measured by ImageJ-Win64 software. The data were obtained from three biological replicates. SPSS v21 (Statistic Package for Social Science) software was used to analyze the significance of the differences. The mean values ± SD from three replicates are presented, and significant differences compared with the control were determined by Student’s *t*-test (* indicates *p* < 0.05; ** indicates *p* < 0.001).

## 5. Conclusions

In this study, we observed that Ae has long, straight, and high-density trichomes, while Al has short, distorted, and low-density trichomes. The second- and third-generation transcriptomic analysis indicated that the expression of the *NAP1* gene was suppressed in Al compared with that in Ae. Additionally, alternative splicing of *AlNAP1* existed in both leaves and fruits of Al but not in that of Ae. The defects of trichome development in *Arabidopsis nap1* mutant were rescued by full-length *AlNAP1* but not by the spliced transcript of *AlNAP1*. *AlNAP1* gene overexpression does not affect trichome density in *nap1* mutant or wild-type plants. The alternative splicing further decreased the level of functional transcript *AlNAP1-FL*, which might cause the short and distorted trichomes in Al. Overall, we revealed that *AlNAP1* mediates trichome development and is a good candidate target for genetic modification of trichome length in kiwifruit.

## Figures and Tables

**Table 1:** Statistical table of three-generation full-length transcriptome clustering of leaves from *A. latifolia* (Al) and *A. eriantha* (Ae).

Samples	Number of Consensus Isoforms	Average Consensus Isoforms Read Length	Number of Polished High-Quality Isoforms	Number of Polished Low-Quality Isoforms	Percent of Polished High-Quality Isoforms (%)	Non-Redundant Transcript Sequences
Al-q	135,307	1844	135,286	21	99.98%	126,102
Ae-q	156,765	1522	156,752	13	99.99%	93,226

Note: Number of consensus isoforms: a consensus sequence derived from clustering; Average consensus isoforms length: Number of HQ isoforms: Number of high-quality (accuracy 0.99) transcripts in a consistent sequence; Percentage of HQ isoforms(%): the percentage of high-quality transcripts in the consistent sequence.

**Table 2:** Statistical table of number of differentially expressed transcripts and genes.

Al_vs_Ae	Total Number	Up-Regulated	Down-Regulated
Differentially expressed transcripts (DETs)	88,270	39,879	48,391
Differentially expressed genes (DEGs)	12,950	5851	7099

All the DETs were mapped to the Ae genome provided by the kiwifruit genome database (http://kiwifruitgenome.org/, accessed on 12 October 2021), and we obtained 12,950 DEGs.

## Data Availability

The raw data of our transcriptomic analysis were uploaded to BioProject Library (https://www.ncbi.nlm.nih.gov/bioproject/, accessed on 18 October 2022), and the accession number is PRJNA889934.

## Supplementary Materials

The following supporting information can be downloaded at: https://www.mdpi.com/article/10.3390/ijms24054481/s1.
