# Supplementary material for: Comparative Transcriptomic Analysis Revealed the Suppression and Alternative Splicing of Kiwifruit (Actinidia latifolia) NAP1 Gene Mediating Trichome Development
Source: Int J Mol Sci. 2023 Feb 24;24(5):4481. doi: 10.3390/ijms24054481 (PMC10003061; doi:10.3390/ijms24054481)
Supplement: Supplementary file 1 [file ijms-24-04481-s001.zip › Supplemental Figures.pdf]

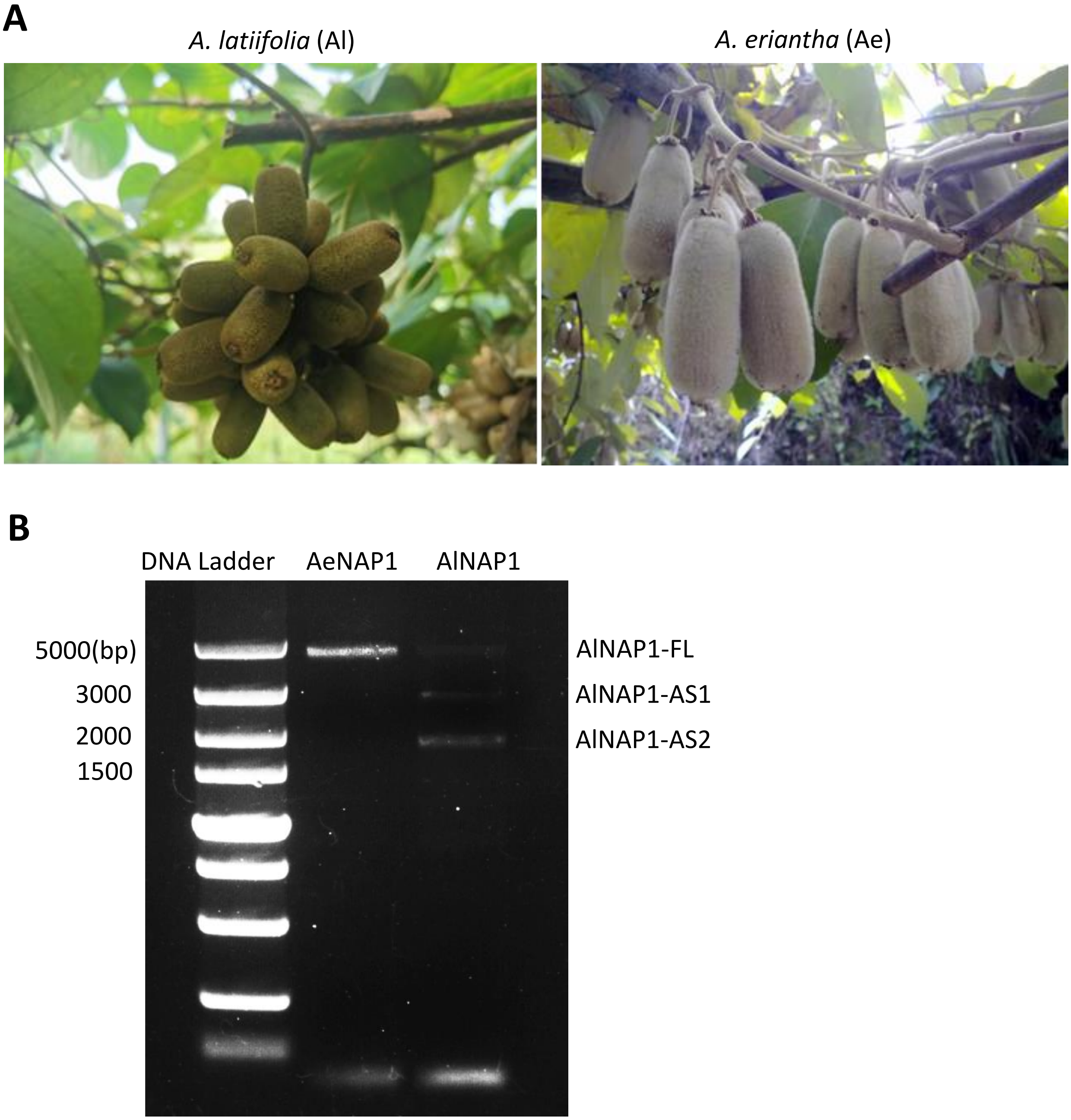

Figure S1. The alterative splicing of *NAP1* exists in Al fruits

(**A**) Morphologic observations of the mature fruits of *A. latifolia* and *A. eriantha*. (**B**) The agarose gel analysis of RT-PCR products of *AeNAP1* and *AlNAP1* including *AlNAP1-FL*, *AlNAP1-AS1*, and *AlNAP1-AS2*. The total RNAs were extracted from the epidermal tissues of young fruits (25 days after full bloom) of both species.

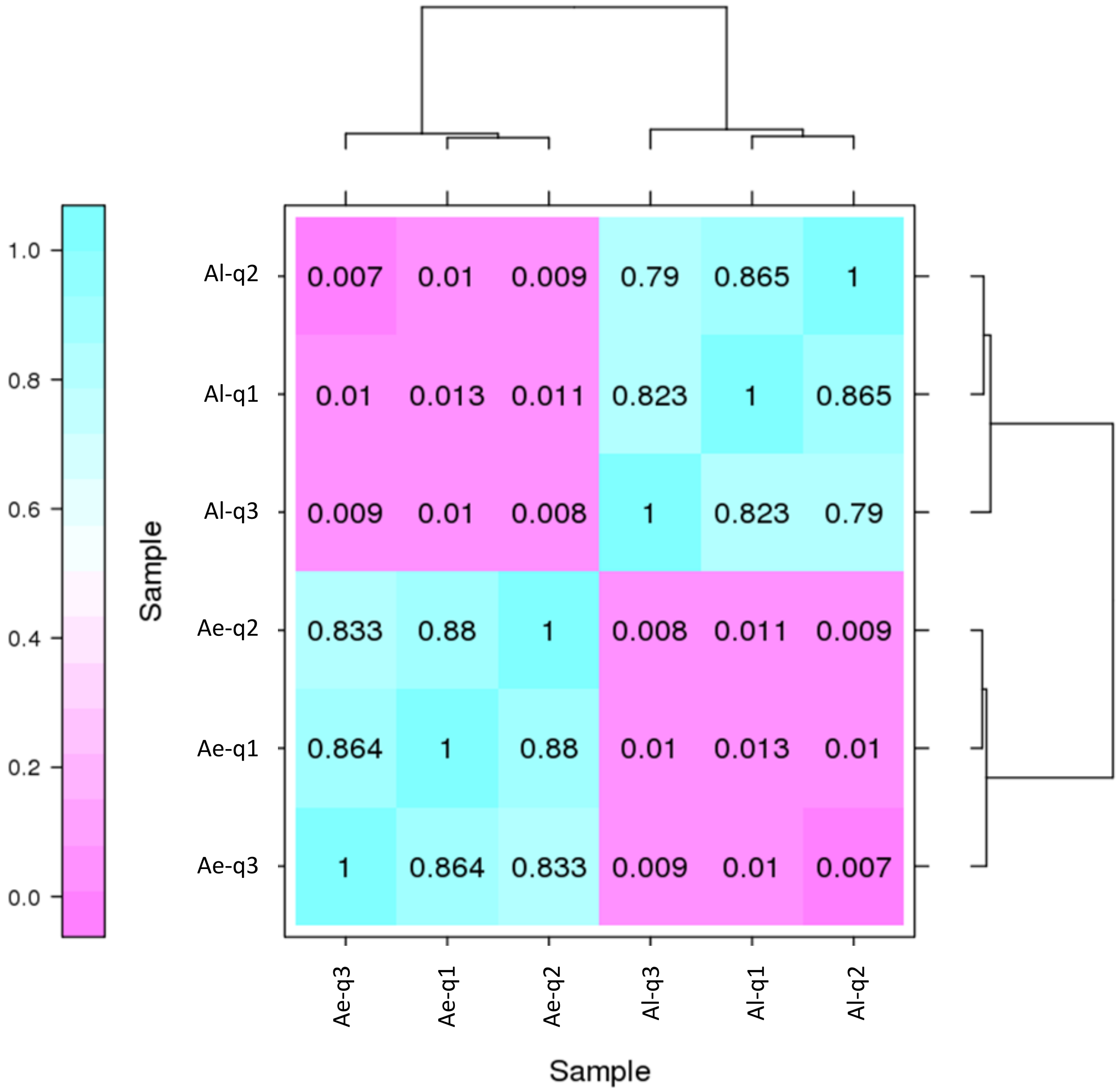

Figure S2. Heat map of expression correlation of pairwise samples

The PCA analysis was performed by using three biological replicates from Al (Al-q1,-q2, and –q3) and Ae (Ae-q1, -q2, and –q3).

```
AeNAP1    MQMAKSRQNYPAQDVFSSAPTAGKSREYEGPARWTEYLGQEMTSQTTSRGSRNGGAEGTVQSLGGSHKGLNMQWVYQLTEVAEGLMAKMYRLNQILDYPDSVGHVFSDAFWKAGVFPNHP   120
AlNAP1    MQMAKSRQNYPAQDAFSSAPTAGKSREYEGPARWTEYLGQEMTSQTTSRGSRNGGSEGTVQSLGGSHKGLNMQWVYQLTEVAEGLMAKMYRLNQILDYPDSVGHVFSDAFWKAGVFPNHP   120
Consensusmqmaksrqnypaqd fssaptagksreyegparwteylgqemtsqttsrgsrngg egtvqslggshkglnmqwvyqltevaeglmakmyrlnqildypdsvghvfsdafwkagvfpnhp

AeNAP1    KICILLSKKFPEHHSKLQLERVDKVALDALNDGAEVHLQSLEPWVQLLLDLMTFREQALRLILDLSSTVITLLPHQNSLILHAFMDLFCAFVRVNLFAEKIPRKMMLQMYNLFHAILHND   240
AlNAP1    KICILLSKKFPEHHSKLQLERVDKVALDALNDGAEVHLQSLEPWVQLLLDLMTFREQALRLILDLSSTVITLLPHQNSLILHAFMDLFCAFVRVNLFAEKIPRKMMLQMYNLFHAILHND   240
Consensuskicillskkfpehhsklqlervdkvaldalndgaevhlqslepwvqllldlmtfreqalrlildlsstvitllphqnslilhafmdlfcafvrvnlfaekiprkmmlqmynlfhailhnd

AeNAP1    RDCDFYHRLVQFIDSYDPPLKGLHEDLNFVSPRIGEVLEAVGPVIFLSTDTRKLRNEGFLSPFHPRYPEILTNSAHPMRAQDLANVTSYREWVLFGYLVCPDELLRVTSIDIALVVLKEN   360
AlNAP1    RDCDFYHRLVQFIDSYDPPLKGLHEDLNFVSPRIGEVLEAVGPVIFLSTDTRKLRNEGFLSPFHPRYPEILTNSAHPMRAQDLANVTSYREWVLFGYLVCPDELLRVTSIDIALVVLKEN   360
Consensusrdcdfyhrlvqfidsydpplkglhedlnfvsprigevleavgpviflstdtrklrnegflspfhprypeiltnsahpmraqdlanvtsyrewvlfgylvcpdellrvtsidialvvlken

AeNAP1    LVLTLHRDEYILLHEDYQLYVLPRILESKKMAKSGRTKQKEADLEYSVAKQVEKMISEVHEQAFLSCDSIHRERRILLKQEIGRMVLFFTDQPSLLAPNIQMVFSALAFAQCEVIWYFQH   480
AlNAP1    LVLTLHRDEYILLHEDYQLYVLPRILESKKMAKSGRTKQKEADLEYSVAKQVEKMISEVHEQAFLSCDSIHRERRILLKQEIGRMVLFFTDQPSLLAPNIQMVFSALAFAQCEVIWYFQH   480
Consensuslvltlhrdeyillhedyqlyvlprileskkmaksgrtkqkeadleysvakqvekmisevheqaflscdsihrerrillkqeigrmvlfftdqpsllapniqmvfsalafaqceviwyfqh

AeNAP1    VGIASSKYKAARMIPVDIDPSDPTIGFLLDGMDHLCCLVRKYITAIRSYALSYLSSCAGRIRFLLGTPGMVALDLDATLKGLFQKIVQHLENIPKPQGESISAITCDLSELRKDWLSILM   600
AlNAP1    VGIASSKYKAARMIPVDIDPSDPTIGFLLDGMDHLCCLVRKYITAIRSYALSYLSSCAGRIRFLLGTPGMVALDLDATLKGLFQKIVQHLENIPKPQGESISAITCDLSELRKDWLSILM   600
Consensusvgiasskykaarmipvdidpsdptigflldgmdhlcclvrkyitairsyalsylsscagrirfllgtpgmvaldldatlkglfqkivqhlenipkpqgesisaitcdlselrkdwlsilm

AeNAP1    IVTSARSSINIRHLEKATVSTGKEGLLSEGNAAYNWSRCVDELESQLSKHGSLKKLYFYHQHLTTVFKNTMFGPEGRPQHCCAWLGVASSFPECASAIVPEEVIKIGRDAVLYVESLIES   720
AlNAP1    IVTSARSSINIRHLEKATVSTGKEGLLSEGNAAYNWSRCVDELESQLSKHGSLKKLYFYHQHLTTVFKNTMFGPEGRPQHCCAWLGVASSFPECASAIVPEEVIKIGRDAVLYVESLIES   720
Consensusivtsarssinirhlekatvstgkegllsegnaaynwsrcvdelesqlskhgslkklyfyhqhlttvfkntmfgpegrpqhccawlgvassfpecasaivpeevikigrdavlyveslies

AeNAP1    IMGGLEGLINILDSEGGFGSLEMQLLPEQAATLMNLTSRISIPSAKSPKGVSGFHFPGFESYPENNSSIKMLEAAMQRLTNLCSVLNDMEPICVLNHVFVLREYMRECILGNFKRRLLAV   840
AlNAP1    IMGGLEGLINILDSEGGFGSLEMQLLPEQAATLMNLTSRISIPSAKSPKGVSGFHFPGFESYPENNSSIKMLEAAMQRLTNLCSVLNDMEPICVLNHVFVLREYMRECILGNFKRRLLAV   840
Consensusimgglegliniildseggfgslemqllpeqaatlmnltsrisipsakspkgvsgfhfpgfesypennssikmleaamqrltnlcsvlndmepicvlnhvfvlreymrecilgnfkrrllav

AeNAP1    LKTDNDLQRPSVLESLIRRHISIVHLAEQHISMDLTQGIREVLLSEAFSGPVSSLHLFDKPAEQQTGSATEAVCNWYIENIIKDMSGAGILFAPLHKCFKSTRPVGGYFADSVTDLRELQ   960
AlNAP1    LKTDNDLQRPSVLESLIRRHISIVHLAEQHISMDLTQGIREVLLSEAFSGPVSSLHLFDKPAEQQTGSATEAVCNWYIENIIKDMSGAGILFAPLHKCFKSTRPVGGYFADSVTDLRELQ   960
Consensuslktdndlqrpsvleslirrhisivhlaeqhismdltqgirevllseafsgpvsslhlfdkpaeqqtgsateavcnwyieniikdmsgagilfaplhkcfkstrpvggyfadsvtdlrelq

AeNAP1    AFVRIFGGYGVDRLDRMMKEHTAALLNCIDTSLRANREVLEAVAGSMHSGDRIERETNMKQIIDMETVVGFCIQAGQTISFDCLLAEAAGAVLEEGAPLIHSLLAGVVKHLPDEIPEKKE  1080
AlNAP1    AFVRIFGGYGVDRLDRMMKEHTAALLNCIDTSLRANREVLEAVAGSMHSGDRIERETNMKQIIDMETVVGFCIQAGQTISFDCLLAEAAGAVLEEGAPLIHSLLAGVVKHLPDEIPEKKE  1080
Consensusafvrifggygvdrldrmmkehtaallncidtslranrevleavagsmhsgdrieretnmkqiidmetvvgfciqagqtisfdcllaeaagavleegaplihsllagvvkhlpdeipekke

AeNAP1    IRRMRRVANSVGVISDHDTEWVRSILEEVGGANDGSWSLLPYFFATFMTSNIWNVTAFNVDTGGFNNNIHCLARCISAVTAGSEFIRLEREHQQRQSFSNGHVSEVLDPDMLNRLSAETS  1200
AlNAP1    IRRMRRVANSVGVISDHDTEWVRSILEEVGGANDGSWSLLPYFFATFMTSNIWNVTAFNVDTGGFNNNIHCLARCISAVTAGSEFIRLEREHQQRQSFSNGHVSEVLDPDMLNRLSAETS  1200
Consensusirrmrrvansvgvisdhdtewvrsileevggandgswsllpyffatfmtsniwnvtafnvdtggfnnnihclarcisavtagsefirlerehqqrqsfsnghvsevldpdmlnrlsaets

AeNAP1    VKSIMQLFVKFSAGIILDSWSESNRSHLVEKLIFLDQLCEVSPYLPRSSLDAHVPYSILRSIYSQFYSNSPSIALALIGVSPRHSPAVSLAASPAIRQPRGDSTPQSSANDSGYFKASST  1320
AlNAP1    VKSIMQLFVKFSAGIILDSWSESNRSHLVEKLIFLDQLCEVSPYLPRSSLDAHVPYSILRSIYSQFYSNSPSIALALIGVSPRHSPAVSLAASPAIRQPRGDSTPQSSANDSGYFKASST  1320
Consensusvksimqlfvkfsagiildswsesnrshlveklifldqlcevspylprssldahvpysilrsiysqfysnspsialaligvsprhspavslaaspairqprgdstpqssandsgyfkasst

AeNAP1    HGQDHLYDAESGSMRSVEHKHRNVRRSGPLDYSSSRKVKFFEGSTSRSTGPSPLPRFAVSRSGPISY                                                      1387
AlNAP1    HGQDHLYDAESGSMRSVEHKHRNVRRSGPLDYSSSRKVKFFEGSTSRSTGPSPLPRFAVSRSGPISY                                                      1387
Consensushgqdhlydaesgsmrsvehkhrnvrrsgpldysssrkvkffegstsrstgpsplprfavsrsgpisy
```

Figure S3. Protein sequence alignment of AeNAP1 and AlNAP1-FL

The protein alignment were performed by ApE software. The identical amino acids were highlighted in dark blue.
